# Malnutrition and its effects in severely injured trauma patients

**DOI:** 10.1007/s00068-020-01304-5

**Published:** 2020-01-23

**Authors:** Suzan Dijkink, Karien Meier, Pieta Krijnen, D. Dante Yeh, George C. Velmahos, Inger B. Schipper

**Affiliations:** 1 Department of Trauma Surgery, Leiden University Medical Center, K6-R, P.O. Box 9600, 2300 Leiden, The Netherlands; 2 Ryder Trauma Center, DeWitt Daughtry Family Department of Surgery, University of Miami Miller School of Medicine, Miami, FL USA; 3 Division of Trauma, Emergency Surgery and Surgical Critical Care, Department of Surgery, Massachusetts General Hospital, Boston, MA USA

**Keywords:** Malnutrition, Trauma, Severe injuries, Nutrition, Adverse outcomes, Polytrauma

## Abstract

**Purpose:**

In hospitalized patients, malnutrition is associated with adverse outcomes. However, the consequences of malnutrition in trauma patients are still poorly understood. This study aims to review the current knowledge about the pathophysiology, prevalence, and effects of malnutrition in severely injured patients.

**Methods:**

A systematic literature review in PubMed and Embase was conducted according to PRISMA-guidelines.

**Results:**

Nine review articles discussed the hypermetabolic state in severely injured patients in relation to malnutrition. In these patients, malnutrition negatively influenced the metabolic response, and vice versa, thereby rendering them susceptible to adverse outcomes and further deterioration of nutritional status. Thirteen cohort studies reported on prevalences of malnutrition in severely injured patients; ten reported clinical outcomes. In severely injured patients, the prevalence of malnutrition ranged from 7 to 76%, depending upon setting, population, and nutritional assessment tool used. In the geriatric trauma population, 7–62.5% were malnourished at admission and 35.6–60% were at risk for malnutrition. Malnutrition was an independent risk factor for complications, mortality, prolonged hospital length of stay, and declined quality of life.

**Conclusions:**

Despite widespread belief about the importance of nutrition in severely injured patients, the quantity and quality of available evidence is surprisingly sparse, frequently of low-quality, and outdated. Based on the malnutrition-associated adverse outcomes, the nutritional status of trauma patients should be routinely and carefully monitored. Trials are required to better define the optimal nutritional treatment of trauma patients, but a standardized data dictionary and reasonable outcome measures are required for meaningful interpretation and application of results.

**Electronic supplementary material:**

The online version of this article (10.1007/s00068-020-01304-5) contains supplementary material, which is available to authorized users.

## Introduction

Malnutrition is an underestimated problem in the general hospitalized population. Estimations up to a 50% prevalence of this condition have been reported, with probably even higher numbers in the critically ill [1–4]. Malnutrition in hospitalized patients is an important factor to consider, because it is associated with adverse outcomes such as prolonged hospital length of stay, increased complications, in-hospital mortality, and healthcare costs [1, 5, 6]. Although most physicians are aware of the risk of malnutrition, half of all malnourished patients are not identified during their hospital stay [7].

In severely injured patients, the relationship between nutritional status and clinical outcome is complicated by the systemic pathophysiological responses to trauma, which may affect, as well as may be affected by, the patient’s nutritional status [8–11]. The impact of nutrition on metabolic changes and clinical outcomes in severely injured trauma patients is therefore unique and complex but remains poorly understood. More insight into these mechanisms may increase awareness of nutritional status in severely injured patients so that both, as classified by the American Society of Parenteral and Enteral Nutrition (A.S.P.E.N), acute disease or injury-related malnutrition and its consequences may be prevented [12]. The purpose of this systematic review is to summarize and evaluate the available literature on: (1) the metabolic effects of malnutrition in severely injured trauma patients, and (2) the incidence/prevalence of malnutrition, the risk of developing malnutrition, and clinical outcomes of malnutrition in severely injured patients.

## Material and methods

### Search strategy

This systematic review was conducted according to the Preferred Reporting Items for Systematic reviews and Meta-Analyses (PRISMA) statement [13]. A systematic literature search was performed in PubMed and Embase with help of an experienced medical librarian in May 2019. The search strategy, provided in Appendix 1, included related terms and synonyms for nutritional status, malnutrition, undernutrition, and adult polytrauma patients.

### Eligibility criteria and article selection

Articles about the pathophysiology and metabolic effects of malnutrition in severely injured trauma patients were considered eligible for inclusion, as well as clinical studies in which the prevalence and/or outcomes of malnutrition in severely injured trauma patients were reported. We did not use a specific definition of malnutrition and severely injured patients because different criteria and assessments tools for both malnutrition and severely injured patients were used in the literature. We selected publications in Dutch, English, French, and German without restriction on publication year. Identified articles were first screened for relevance based on title and abstract. Articles without full-text were not included; the majority (56%) of these were outdated (> 20 years old). The full-text of potentially eligible articles were read before inclusion in the review. The reference lists of the included articles were screened for additional literature. Expert opinions, conference papers and letters to the editor were excluded. Selected articles were grouped by topic: (1) articles describing the metabolic response of malnutrition in severely injured trauma patients, and (2) clinical cohort studies describing the prevalence of malnutrition and its association with clinical outcomes in severely injured trauma patients during hospital admission.

### Data extraction and risk of bias assessment

#### Metabolic effects; reviews

All selected articles about the pathophysiology and metabolic effects of malnutrition in severely injured trauma patients were review articles. Data on pathophysiology and metabolic effects of malnutrition in these articles were summarized and combined in a model. The methodological quality of the included review articles could not be assessed according to the AMSTAR tool or equivalent as suggested by the PRISMA-guidelines [14, 15], because none of these articles were systematic reviews.

#### Incidence/prevalence and outcomes of malnutrition; cohort studies

Patients’ age and gender, reported prevalence of malnutrition, type of nutritional assessment tool, and reported clinical outcomes were extracted from the selected cohort studies. Authors were contacted for more detailed information on the severely injured and geriatric patients groups in their studies, however none of the contacted authors responded to our query. Due to the inconsistent and different assessment tools and reported outcomes, the extracted data of these studies could not be pooled.

The risk of different types of bias of the included cohort studies was assessed using the ‘Methodological index for non-randomized studies’ (MINORS criteria) on a 3-point scale ranging from 2 (reported and adequate) to 0 (not reported) [16].

Article selection, data extraction and assessment of methodological quality were performed independently by the first two authors. Disagreement was resolved by discussion.

## Results

### Selection of articles

**Fig. 1:**
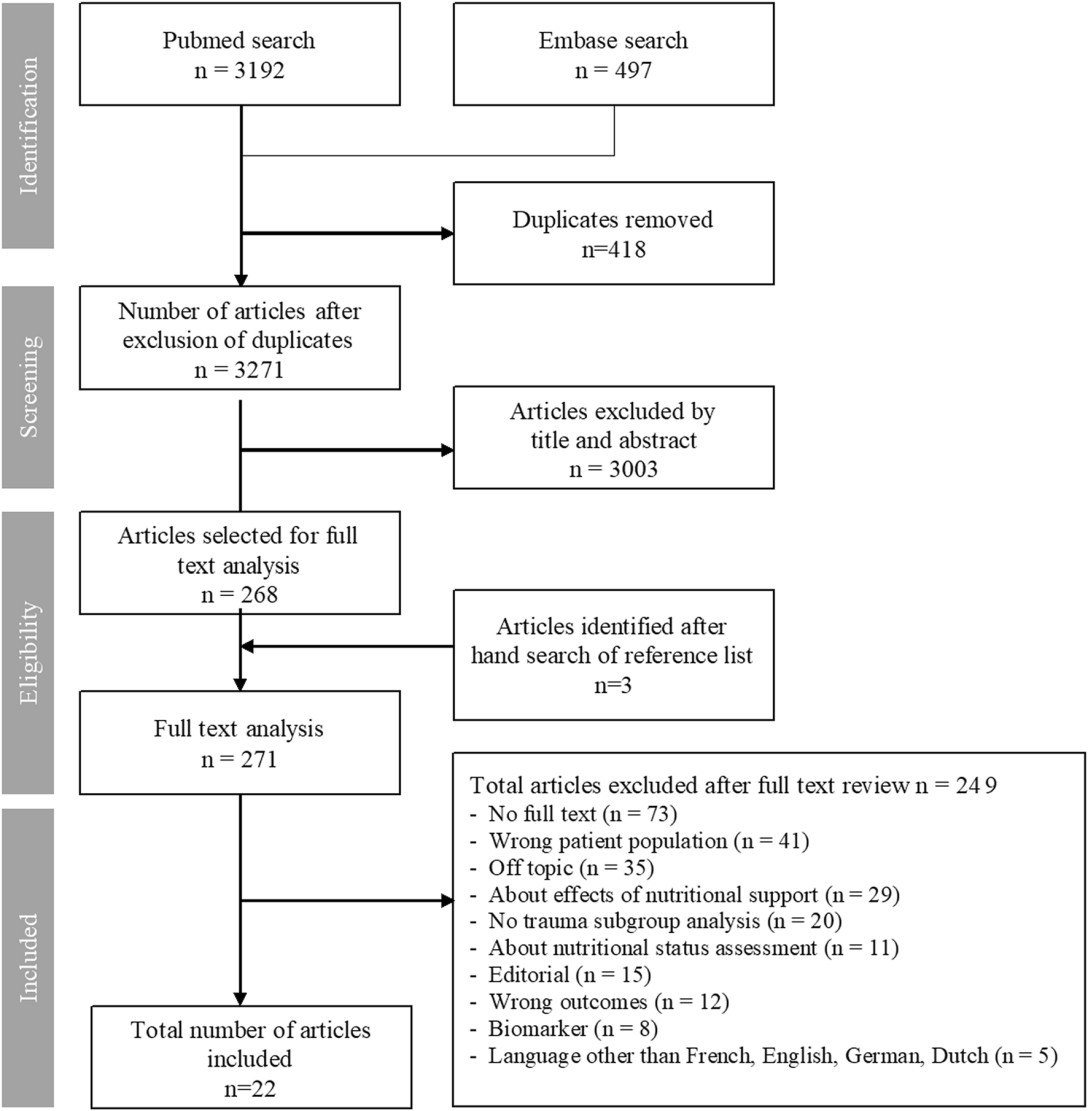
Studies resulting from literature search with reasons for in-/exclusion

The search yielded 3689 articles. After removing 418 duplicates, 3271 articles were screened for eligibility. Seventy-three articles were excluded because no full-text was available. Twenty articles fulfilled the inclusion criteria and were included in this review. Two more studies were included based on hand search of the reference lists (Fig. 1).

#### Metabolic effects

Nine articles, published between 1968 and 2011, were non-systematic reviews about the altered metabolic state of severely injured trauma patients [11, 17–24]. All these articles discussed a specific part of the metabolic response in relationship to malnutrition.

#### Incidence/prevalence and outcomes of malnutrition

Thirteen cohort studies about the prevalence of malnutrition in severely injured trauma patients were found, published between 1987 and 2019 (Table 1) [10, 25–33]. Outcomes were reported in ten articles, of which six described the general trauma population with severe injuries [10, 25, 31, 33–35], four described the geriatric trauma population [26, 27, 30, 34]. The risk of bias in the included cohort studies was generally low (Table 2).

**Table 1:** Included cohort studies about prevalence of malnutrition and its effects on outcomes in trauma patients in this review (*n* = 10)

References	Year	Country	Design	*n*	Male (%)	Age in years	Nutritional assessment tool	Prevalence MN on admission (unless indicated otherwise) (%)	ARM (%)	Mortality	Length of stay in days	Complications
Wilson et al. [36]	2019	United States	Prospective cohort study	377	50.9	73.70^m^ ± SD 12.73	Visceral proteins	Hypoalbuminaemia: 17.5 Low TLC: 62.3	–	Hypoalbuminaemia: OR2.22 95% CI 1.26–3.92 TLC: n/a	Hypoalbuminaemia: *r* = − 0.14, *p* = 0.024 TLC:n/a	≥ 1 adverse event associated with MN, *p* < 0.001
Wilson et al. [34]	2019	United States	Retrospective cohort study	5673	43.8	46.69^m^ ± SD 13.62	Visceral proteins	Hypoalbuminaemia: 29.6		WN: 0.4%, MN: 3.2%, RR 4.86, 95% CI 2.66–8.87	WN: 3.57 (± SD 5.0) MN: 7.5 (± SD 10.45) *p* < 0.001	≥ 1 adverse event RR 1.46 95% CI 1.30–1.64; Sepsis RR 1.99 95% CI 1.03–3.86; Unplanned intubation RR 2.95 95% CI 1.49–5.84; Reoperation RR 1.52 95% CI 1.11–2.07; Readmission RR 2.0 95% CI 1.55–2.57
Wintermeyer et al. [35]	2019	Germany	Prospective cohort study	1642	–	57.8^m^ ± SD 16.6	NRS	–	Overall: 18.3 Geriatric trauma: 35.6	–	–	≥ 1 adverse event associated with ARM, *p* < 0.01; quality of life negatively associated with ARM, *p* < 0.01
Ihle et al. [33]	2017	Germany	Prospective cohort study	521	56.2	53.9^m^ ± SD 18.1	NRS	–	19.2	–	NRS ≥ 3 (ARM): 16 ± SD 12 NRS < 3 (WN): 11 ± SD 10	≥ 1 adverse event associated with ARM, *p* < 0.001
Müller et al. [26]	2017	Switzerland	Non-comparative prospective cohort study	169	42.6	79.7^m^ ± SD 6.5	MNA	7.1	49.1	–	–	–
Goisser et al. [27]	2015	Germany	Non-comparative retrospective cohort study	97	20.6	84.0^m^ ± SD 5.0	MNA (long form)	17.0	38.0	WN:13%, ARM: 21%, MN: 0%, *p* = 0.120	WN:11^med^ IQR 10–16, ARM:12^med^ IQR 9–17, MN:10^med^ IQR 7–15, *p* = 0.388	WN: 86%, ARM: 97%, MN: 100%, *p* = 0.095
Chakravarty et al. [28]	2013	India	Non-comparative prospective cohort study	61	78.7	–	SGA	15.0	–	–	–	–
Banks et al. [29]	2010	Ireland	Non-comparative prospective cohort study	30	37.0	78.5^med^ IQR 68–85	MNA	–	60.0	–	–	–
Dhandapani et al. [25]	2007	India	Non-comparative prospective cohort study	61	92.0	35.4^m^ ± SD not reported	Anthropometric measurements	Clinical features of pedal edema, cheilosis, skeletal prominence, xerosis, gum bleed: Week 1: 45.0 Week 3: 76.0		–	–	–
Goiburu et al. [10]	2006	Paraguay	Non-comparative prospective cohort study	161	94.0	27.0^med^ IQR 14–92	SGA	40.0	–	RR 4; 95% CI 1–15	> 14 days RR 2.3; 95% CI 1.2–4.7	RR 2.9; 95% CI 1.4–5.8
Compan et al. [30]	1999	France	Non-comparative prospective cohort study	299	33.0	82.9^m^ ± SD 7.0	MNA	24.7	–	–	Longer stay associated with MN, p not reported	Death during hospitalization associated with MN, *p* < 0.0001
McClave et al. [31]	1992	United States	Non-comparative prospective cohort study	–	–	–	Visceral proteins Anthropometric measurements	Low visceral proteins (albumin, transferrin, TLC): 17.6%, Underweight: 20.6%, Mix: 15.6%		OR 4.04; *p* < 0.05	OR 1.29; *p* < 0.05	Sepsis: OR 2.64; *p* < .05; Nosocomial infections: OR 2.26; *p* < 0.05
Kaufman et al. [32]	1987	United States	Non-comparative prospective cohort study	76	–	–	Visceral proteins Anthropometric measurements	Albumin, transferrin, TLC, and others: not defined	-	–	–	–

*MN* Malnourished, *ARM* At risk for malnutrition, *WN* Well−nourished, *NRS* Nutritional Risk Screening, *MNA* Mini Nutritional Assessment, *SGA* Subjective Global Assessment scale, *TLC* Total lymphocyte count, *SD* Standard deviation, *IQR* Interquartile range, *RR* relative risk, *C*: confidence interval, *OR* adjusted odds ratio

*n refers to the number of trauma patients in the study

m mean

med median

**Table 2:** Risk of Bias in the included cohort studies according to Methodological Index for Non-Randomized Studies (MINORS) criteria [16]

	Wilson et al. [36]	Wilson et al. [34]	Wintermeyer et al. [35]	Ihle et al. [33]	Müller et al. [26]	Goisser et al. [27]	Chakravarty et al. [28]	Banks et al. [29]	Dhandapani et al. [25]	Goiburu et al. [10]	Compan et al. [30]	McClave et al. [31]	Kaufman et al. [32]
1. Clearly stated aim	2	2	2	2	2	2	2	2	2	2	2	1	2
2. Inclusion of consecutive patients	2	2	2	2	2	2	2	2	2	2	2	0	0
3. Prospective collection of data	2	2	2	2	2	2	0	2	2	2	2	2	0
4. Endpoints appropriate to aim of study	2	2	2	2	2	2	2	2	2	2	2	0	2
5. Unbiased assessment of study endpoints	2	2	2	0	2	0	0	2	2	0	1	0	0
6. Follow-up period appropriate to aim of study	2	2	2	2	2	2	2	2	2	2	2	2	2
7. < 5% lost to follow-up	2	1	2	2	0	2	2	1	1	1	0	2	1
8. Prospective calculation of study size	0	0	0	0	2	2	2	1	1	1	1	0	0
Total	14	13	14	12	14	14	12	14	14	12	12	7	7

Criteria were scored 2 (reported and adequate), 1 (reported but inadequate) or 0 (not reported), with a maximum total score of 16

### Malnutrition and the metabolic response in severely injured patients

**Fig. 2:**
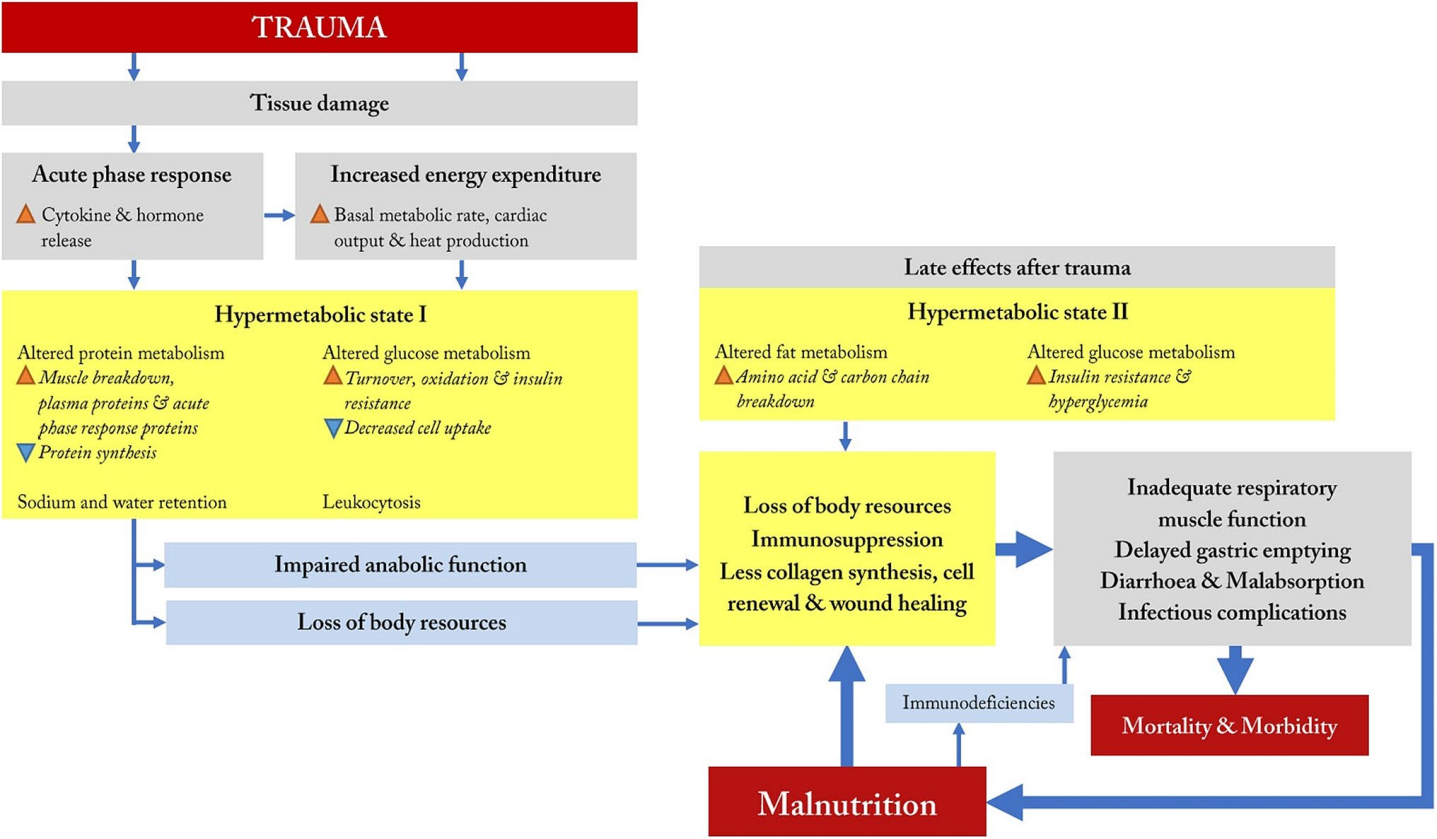
Model of effects of the hypermetabolic state and malnutrition in severely injured patients

The pathophysiological processes and metabolic effects of malnutrition in severely injured patients described in the nine review articles are summarized in Fig. 2. Essentially, the reviews describe the combination of a prolonged and/or disturbed metabolic response following traumatic injury and the negative influence of malnutrition upon this response that leads to a vicious circle of further deterioration of the nutritional- and health status and the metabolic response [20, 21, 24].

After severe trauma, burns, or infection, a universal acute phase response is seen, characterized by a predominantly hypermetabolic catabolic state [11, 17–20, 23, 24]. Although this acute phase response seems to be essential for recovery, a maladaptive prolonged and/or disturbed metabolic response is associated with complications, morbidity and mortality [24].

The initial post-injury state in severely injured patients, caused by tissue damage, is characterized by an acute phase response and increased energy expenditure. Released cytokines (e.g., interleukins and tumor necrosis factor-α) together with post-injury released hormones (including epinephrine, cortisol, and glucagon), act as catabolic stimulants [11, 17, 18, 23]. Depending upon the severity of injury, energy expenditure increases by 20–50% in trauma patients compared to patients after elective surgery (Fig. 2).

The combination of this acute phase response and increased energy expenditure after trauma leads to a hypermetabolic state (Fig. 2; “Hypermetabolic state I”). This hypermetabolic state alters protein metabolism, leading to increased muscle protein mobilization for energy, and decreased protein synthesis leading to catabolism [19, 22]. In addition, leukocytosis, changes in the glucose metabolism, and retention of sodium and water are seen [18]. These biochemical adaptations are distinctive for severely injured patients, and increase the susceptibility of the trauma patient for developing malnutrition.

Recovery of the trauma patient is threatened by the combination of increased loss of body resources and the prolonged and/or disturbed hypermetabolic state. This state is characterized by impaired anabolic function characterized by continued muscle protein breakdown and ongoing elevated energy expenditure remains high (Fig. 2) [18, 20, 23].

Additionally, between three to seven days post-injury, severely injured trauma patients developed increased lipid metabolism, insulin resistance, and hyperglycemia (Fig. 2; “Hypermetabolic state II”) [23]. Due to this insulin resistance, patients develop a glucose deficit, causing the body to oxidize branched carbon chains from amino acids for energy production [17, 18, 22, 23]. This altered fat metabolism further contributes to significant breakdown in amino acids and body protein stores, reflected by a negative nitrogen balance [24]. It has been suggested that 10–15% of the weight loss in trauma patients is a consequence of depleting normal protein stores [22]. However, severely malnourished patients are unable to increase their protein turnover, which has been associated with a higher risk of mortality, with inefficient wound healing, and less cell renewal [24].

One clinically relevant consequence of muscle breakdown is seen in respiratory muscle inadequacy and the associated prolonged ventilator dependency, pneumonia, and subsequent risk of mortality [17, 22]. Immunosuppression and the cytokine cascade can lead to a functionally impaired gastrointestinal tract, contributing to delayed gastric emptying, diarrhea, and malabsorption [17, 24]. These consequences of the distinctive metabolic response following traumatic injuries not only increase mortality and morbidity rates, but also potentiate deterioration of the nutritional status [17, 22, 24]. Once malnutrition has developed, the circle is complete, as it negatively influences the metabolic response, leading to relative immunodeficiency, such as an impaired white blood cell function, decreased T-cell function and anti-body and complex formation [17, 22, 24]. This again makes the patient particularly susceptible to infectious complications and further loss of body resources [18, 20, 22, 24].

### Prevalence of malnutrition and its association with patient outcomes

Thirteen cohort studies reported on the prevalence of malnutrition in severely injured trauma patients (Table 1). The prevalence of malnutrition ranged from 7 to 76% in trauma patients in general [10, 25–32, 34–36].

Six studies specifically reported the prevalence of malnutrition on geriatric patients admitted with traumatic injuries. On admission, 7–62.6% of the geriatric trauma patients were malnourished and 35.6–60% were at risk for malnutrition according to the Mini Nutritional Assessment (MNA) tool, Nutritional Risk Screening (NRS 2002) tool and serum biomarkers (visceral proteins) [26, 27, 29, 30, 35, 36]. Dhandapani et al. also examined the development of malnutrition during hospital stay and showed an increase in prevalence from 45% in the first week of hospital admission to 76% in the third week [25].

Ten studies reported on clinical outcomes, such as mortality, hospital length of stay, quality of life, and complications associated with malnutrition, six in the general trauma population [10, 25, 31, 33–35] and four in the geriatric trauma population specifically [26, 27, 30, 36] (Table 1). Malnutrition was associated with higher morbidity, delayed mobilization both after conservative and operative treatment, higher in-hospital mortality, prolonged hospital length of stay, reoperation and readmissions [10, 31, 33–35]. In malnourished patients with traumatic brain injuries, neurological outcome after 6 months was less favorable, classified as death, persistent vegetative state or severe disability according to the Glasgow Outcome Scale (adjusted odds ratio 12.5; 95% confidence interval 2.6–61.0) compared to well-nourished patients [25].

Geriatric patients at risk for malnutrition or suffering from malnutrition had more often cognitive impairments, infectious complications, depressive symptoms, comorbidity, less amelioration of their nutritional status, a higher prevalence of frailty and suspected sarcopenia than well-nourished patients [26, 27, 29, 36]. Two studies observed no differences in length of stay, readmission rates and mortality rates [27, 30], while one cohort study observed a negative associations between malnutrition and these aforementioned outcomes [36]. Malnutrition seemed to have an influence on health-related quality of life, as malnourished geriatric trauma patients suffered more often than well-nourished geriatric trauma patients from irreversible loss of independency and worse physical, mental, and cognitive health after trauma [27].

## Discussion

It is universally recognized that baseline “malnutrition” is a risk factor for worse clinical outcomes and that nutritional adequacy is important in component of the complex multidisciplinary care of severely injured patients. Yet, our comprehensive review of the published literature reveals that the evidence-base for these commonly held tenets is surprisingly sparse, outdated, and frequently of low-quality. The practice of critical care is rapidly changing, and many of the “landmark” studies in metabolism have been performed prior to recent treatment paradigm shifts in blood transfusion, fluid administration, sedation interruption, and ventilator management, to name but a few. Very few actual scientific studies have been conducted in the modern era of critical care and the literature is marked by heterogeneity in assessment tools, assessment times, interventions, and outcomes. It is thus impossible to quantitatively synthesize the literature. As such, we attempt to qualitatively synthesize the literature and offer suggestions to improve future studies in the field of nutrition in polytrauma patients.

The metabolic changes after trauma are distinctive and complex and make trauma patients more susceptible for developing malnutrition. Second, in these patients a vicious circle is set in motion by malnutrition, leading to further deterioration of the nutritional- and health status. The results of the review also underline the importance of early malnutrition recognition and intervention to prevent further deterioration.

The prevalence of malnutrition varied widely in the selected studies and depends upon the way in which malnutrition was defined and measured. Malnutrition has been defined in various ways [37, 38], due to the lack of a gold standard for diagnosing malnutrition. Sánchez-Rodriquez et al. compared two different tools in the same patient population and demonstrated little agreement on the presence of malnutrition [39]. To uniformly diagnose malnutrition and determine its prevalence, a generally accepted standard definition and validated assessment tool is required. According to the American Society for Parenteral and Enteral Nutrition (A.S.P.E.N.) clinical guidelines there are currently 11 screening tools for assessing the risk of malnutrition, and two validated tools for diagnosing malnutrition [40]. We therefore conclude that there is a need for a simple, valid, and generally accepted method for the assessment of nutritional status in hospitalized patients, which facilitates early identification and treatment of malnutrition but also determination of the prevalence of malnutrition in this patient population. At present, the best candidate for this assessment tool is the Nutrition Risk in the Critically Ill (NUTRIC) score [41–44]. The NUTRIC-score was specifically developed to identify critically ill patients who would benefit from nutritional support [45] and has been well-validated for several important outcomes, such as ICU length of stay, ventilator-free days, and mortality. The NUTRIC-score is currently recommend by the Society of Critical Care Medicine (SCCM) and American Society for Parenteral and Enteral Nutrition (A.S.P.E.N.) 2016 guidelines to assess the nutritional status [42, 44, 46].

The detection of malnutrition in an early phase provides the clinician with the opportunity to intervene and attempt to prevent further deterioration of the nutritional status. The literature suggests that only a fraction of malnourished hospitalized patients receive timely nutritional support to prevent nutritional status decline [47]. Kondrup et al. suggested that hospitalized patients often receive less than the optimal amount of nutrition due to lack of awareness and sub-optimal education of the medical staff [48]. This is supported by Dupertuis et al. who showed that if patients’ nutritional requirements were not met, this was often due to other reasons than illness or treatment, such as inadequate meal services [49]. Improved training of medical staff in recognizing and treating malnutrition is needed to create more awareness for the underestimated problem of malnutrition.

Although it seems intuitively obvious that the problem can be easily solved by providing the patients with the appropriate amount of calories and proteins, there is no strong evidence that increasing nutrient delivery improves clinical outcomes [46, 50]. Recent high-profile trials even suggest that intensive medical nutrition therapy, (receiving > 75% of estimated daily energy and protein requirements) is associated with higher mortality and that permissive underfeeding does not worsen clinical outcomes in patients [51–53]. However, it is important to note that the majority of subjects enrolled in these trials were not malnourished at baseline. Large observational trials have demonstrated that only patients with a BMI < 25 or > 35 seem to benefit from increased nutrition delivery [54, 55]. Additionally, in both the EDEN- and PermiT-trials, the “full” nutrition group did not achieve currently recommended doses of calories nor protein. In all those studies, severely injured trauma patients only comprised a very small percentage of enrolled subjects. The potential benefit for early enteral nutrition in trauma patients is still debatable. Included studies are often of low-quality, heterogenous and included a small study population, and still leaves questions unanswered, such as composition of the enteral nutrition used, nutritional goal, use of supplemental parenteral nutrition and adding supplements to the formula [56]. Thus, our current understanding about the role of malnutrition in trauma patients is built upon a thin evidence-base and most of current practice is extrapolated from studies in non-surgical and non-trauma patients.

## Limitations

A limitation of our systematic review on pathophysiological processes and metabolic changes after severe trauma is that this part was based on reviews (some published more than two decades ago) and mostly not performed according to the currently applied systematic review guidelines. We do consider this knowledge to be important, although we do acknowledge that there are currently new insights being developed. The clinical studies on prevalence and outcomes of malnutrition were small cohort studies, with ill-defined patient populations and great variation in outcomes. Other limitations are that our review was incomplete as the full-texts of 73 identified articles could not be retrieved, and that we imposed language restrictions.

## Recommendations for future research

Based on the overall findings uncovered by this systematic review, we believe that future research on nutrition-related research in severely injured trauma patients should incorporate the following: First, as already mentioned, we recommend the widespread use of a uniform, simple, and validated risk stratification score for which we would recommend the NUTRIC-score. Second, recognizing that traditional biomarkers for monitoring nutritional status (i.e., albumin and prealbumin) are strongly influenced by the acute phase response, we recommend that a C-reactive protein be measured concomitantly to give information about the inflammation status to show that albumin and prealbumin are more related to the acute phase response than nutritional status [57, 58]. In addition, future research should focus on finding new biomarkers that are less affected by the acute phase response and pre-existent comorbidities [57]. Third, we recommend that future studies should carefully consider baseline nutritional status when defining inclusion/exclusion criteria and consider stratifying interventions according to malnourishment. Fourth, we recommend that clinical outcomes be carefully chosen to be reasonably affected by nutritional interventions, clinically relevant, and that time points be standardized across research studies [59].

## Conclusion

Despite widespread belief about the importance of nutrition in severely injured patients, the quantity and quality of available evidence is surprisingly sparse, low-quality, and outdated. Nutritional assessment and ongoing monitoring is hampered by low prioritization and heterogeneous, unvalidated tools. However, based on the malnutrition-associated adverse outcomes, the nutritional status of severely injured trauma patients should be routinely and carefully monitored. This review shows that the combination of a prolonged and/or disturbed metabolic response following severe traumatic injuries that negatively influences the nutritional status and the negative influence of malnutrition upon this response, leads to a vicious circle of further deterioration of the nutritional- and health status. Additional trials are required to better define the optimal nutritional treatment of severely injured patients, but a standardized data dictionary and reasonable outcome measures are required for meaningful interpretation and application of results.

## Electronic supplementary material

Below is the link to the electronic supplementary material.

Supplementary file1 (DOCX 16 kb)

## References

[1] Mogensen KM, Robinson MK, Casey JD, Gunasekera NS, Moromizato T, Rawn JD (2015). Nutritional status and mortality in the critically Ill. Crit Care Med.

[2] Barker LA, Gout BS, Crowe TC (2011). Hospital malnutrition: prevalence, identification and impact on patients and the healthcare system. Int J Environ Res Public Health.

[3] Kruizenga HM, Van Tulder MW, Seidell JC, Thijs A, Ader HJ, Van Bokhorst-de van der Schueren MA (2005). Effectiveness and cost-effectiveness of early screening and treatment of malnourished patients. Am J Clin Nutr.

[4] Thomas JM, Isenring E, Kellett E (2007). Nutritional status and length of stay in patients admitted to an Acute Assessment Unit. J Hum Nutr Diet.

[5] Correia MI, Waitzberg DL (2003). The impact of malnutrition on morbidity, mortality, length of hospital stay and costs evaluated through a multivariate model analysis. Clin Nutr (Edinburgh, Scotland).

[6] Amaral TF, Matos LC, Tavares MM, Subtil A, Martins R, Nazare M (2007). The economic impact of disease-related malnutrition at hospital admission. Clin Nutr (Edinburgh, Scotland).

[7] Kruizenga HM, Wierdsma NJ, van Bokhorst MA, de van der S, Haollander HJ, Jonkers-Schuitema CF (2003). Screening of nutritional status in The Netherlands. Clin Nutr (Edinburgh, Scotland).

[8] Clendenen N, Nunns GR, Moore EE, Reisz JA, Gonzalez E, Peltz E (2017). Hemorrhagic shock and tissue injury drive distinct plasma metabolome derangements in swine. J Trauma Acute Care Surg.

[9] Trunkey DD (1983). Trauma. Sci Am Lancet.

[10] Goiburu ME, Goiburu MM, Bianco H, Diaz JR, Alderete F, Palacios MC (2006). The impact of malnutrition on morbidity, mortality and length of hospital stay in trauma patients. Nutr Hosp.

[11] Rogobete AF, Sandesc D, Papurica M, Stoicescu ER, Popovici SE, Bratu LM (2017). The influence of metabolic imbalances and oxidative stress on the outcome of critically ill polytrauma patients: a review. Burns Trauma.

[12] Jensen GL, Mirtallo J, Compher C, Dhaliwal R, Forbes A, Grijalba RF (2010). Adult starvation and disease-related malnutrition: a proposal for etiology-based diagnosis in the clinical practice setting from the International Consensus Guideline Committee. JPEN J Parenter Enteral Nutr.

[13] Moher D, Liberati A, Tetzlaff J, Altman DG (2009). Preferred reporting items for systematic reviews and meta-analyses: the PRISMA statement. PLoS Med.

[14] Shea BJ, Grimshaw JM, Wells GA, Boers M, Andersson N, Hamel C (2007). Development of AMSTAR: a measurement tool to assess the methodological quality of systematic reviews. BMC Med Res Methodol.

[15] Shea BJ, Hamel C, Wells GA, Bouter LM, Kristjansson E, Grimshaw J (2009). AMSTAR is a reliable and valid measurement tool to assess the methodological quality of systematic reviews. J Clin Epidemiol.

[16] Slim K, Nini E, Forestier D, Kwiatkowski F, Panis Y, Chipponi J (2003). Methodological index for non-randomized studies (minors): development and validation of a new instrument. ANZ J Surg.

[17] Burns HJ (1988). The metabolic and nutritional effects of injury and sepsis. Bailliere's Clin Gastroenterol.

[18] Chiolero R, Revelly JP, Tappy L (1997). Energy metabolism in sepsis and injury. Nutrition (Burbank, Los Angeles County, Calif).

[19] Cuthbertson DP, Tilstone WJ (1968). Nutrition of the Injured. Am J Clin Nutr.

[20] Gusberg RJ (1986). The multiple trauma victim: a nutritional cripple. Yale J Biol Med.

[21] Hoffmann M, Rueger JM (2011). Nutritional status influences trauma outcome. Der Unfallchirurg.

[22] Kinney JM, Elwyn DH (1983). Protein metabolism and injury. Annu Rev Nutr.

[23] Ryan NT (1976). Metabolic adaptations for energy production during trauma and sepsis. Surg Clin North Am.

[24] Soeters PB, Grimble RF (2009). Dangers, and benefits of the cytokine mediated response to injury and infection. Clin Nutr (Edinburgh, Scotland).

[25] Dhandapani SS, Manju D, Sharma BS, Mahapatra AK (2007). Clinical malnutrition in severe traumatic brain injury: factors associated and outcome at 6 months. Indian J Neurotrauma.

[26] Muller FS, Meyer OW, Chocano-Bedoya P, Schietzel S, Gagesch M, Freystaetter G (2017). Impaired nutritional status in geriatric trauma patients. Eur J Clin Nutr.

[27] Goisser S, Schrader E, Singler K, Bertsch T, Gefeller O, Biber R (2015). Malnutrition according to mini nutritional assessment is associated with severe functional impairment in geriatric patients before and up to 6 months after hip fracture. J Am Med Dir Assoc.

[28] Chakravarty C, Hazarika B, Goswami L, Ramasubban S (2013). Prevalence of malnutrition in a tertiary care hospital in India. Indian J Crit Care Med.

[29] Banks LN, Byrne N, Henari S, Morris S, McElwain JP (2010). Nutritional status of elderly trauma patients presenting to a South Dublin Teaching Hospital. Eur Geriatr Med.

[30] Compan B, di Castri A, Plaze JM, Arnaud-Battandier F (1999). Epidemiological study of malnutrition in elderly patients in acute, sub-acute and long-term care using the MNA. J Nutr Health Aging.

[31] McClave SA, Mitoraj TE, Thielmeier KA, Greenburg RA (1992). Differentiating subtypes (hypoalbuminemic vs marasmic) of protein-calorie malnutrition: incidence and clinical significance in a university hospital setting. JPEN J Parenter Enteral Nutr.

[32] Kaufman HH, Bretaudiere JP, Rowlands BJ, Stein DK, Bernstein DP, Wagner KA (1987). General metabolism in head injury. Neurosurgery.

[33] Ihle C, Freude T, Bahrs C, Zehendner E, Braunsberger J, Biesalski HK (2017). Malnutrition—An underestimated factor in the inpatient treatment of traumatology and orthopedic patients: a prospective evaluation of 1055 patients. Injury.

[34] Wilson J, Lunati M, Grabel Z, Staley C, Schwartz AM, Schenker M (2019). Hypoalbuminemia is an independent risk factor for 30-day mortality, postoperative complications, readmission, and reoperation in the operative lower extremity orthopedic trauma patient. J Orthop Trauma.

[35] Wintermeyer E, Ihle C, Ehnert S, Schreiner AJ, Stollhof L, Stockle U (2019). Assessment of the influence of diabetes mellitus and malnutrition on the postoperative complication rate and quality of life of patients in a clinic focused on trauma surgery. Zeitschrift fur Orthopadie und Unfallchirurgie.

[36] Wilson JM, Boissonneault AR, Schwartz AM, Staley CA, Schenker ML (2019). Frailty and malnutrition are associated with inpatient postoperative complications and mortality in hip fracture patients. J Orthop Trauma.

[37] Kinosian B, Jeejeebhoy KN (1995). What is malnutrition? Does it matter?. Nutrition (Burbank, Los Angeles County, Calif).

[38] Lochs H, Allison SP, Meier R, Pirlich M, Kondrup J, Schneider S (2006). Introductory to the ESPEN guidelines on enteral nutrition: terminology, definitions and general topics. Clin Nutr.

[39] Sanchez-Rodriguez D, Marco E, Ronquillo-Moreno N, Maciel-Bravo L, Gonzales-Carhuancho A, Duran X (2018). ASPEN-AND-ESPEN: a postacute-care comparison of the basic definition of malnutrition from the American Society of Parenteral and Enteral Nutrition and Academy of Nutrition and Dietetics with the European Society for Clinical Nutrition and metabolism definition. Clin Nutr (Edinburgh, Scotland).

[40] Mueller C, Compher C, Ellen DM (2011). A.S.P.E.N. clinical guidelines: nutrition screening, assessment, and intervention in adults. JPEN J Parenter Enter Nutr.

[41] Heyland DK, Dhaliwal R, Jiang X, Day AG (2011). Identifying critically ill patients who benefit the most from nutrition therapy: the development and initial validation of a novel risk assessment tool. Crit Care (London, England).

[42] Mukhopadhyay A, Henry J, Ong V, Leong CS, Teh AL, van Dam RM (2017). Association of modified NUTRIC score with 28-day mortality in critically ill patients. Clin Nutr (Edinburgh, Scotland).

[43] Mendes R, Policarpo S, Fortuna P, Alves M, Virella D, Heyland DK (2017). Nutritional risk assessment and cultural validation of the modified NUTRIC score in critically ill patients—-A multicenter prospective cohort study. J Crit Care.

[44] de Vries MC, Koekkoek WK, Opdam MH, van Blokland D, van Zanten AR (2018). Nutritional assessment of critically ill patients: validation of the modified NUTRIC score. Eur J Clin Nutr.

[45] Coltman A, Peterson S, Roehl K, Roosevelt H, Sowa D (2015). Use of 3 tools to assess nutrition risk in the intensive care unit. JPEN J Parenter Enter Nutr.

[46] McClave SA, Taylor BE, Martindale RG, Warren MM, Johnson DR, Braunschweig C (2016). Guidelines for the provision and assessment of nutrition support therapy in the adult critically Ill patient: Society of Critical Care Medicine (SCCM) and American Society for Parenteral and Enteral Nutrition (A.S.P.E.N.). JPEN J Parenter Enter Nutr.

[47] Norman K, Pichard C, Lochs H, Pirlich M (2008). Prognostic impact of disease-related malnutrition. Clin Nutr (Edinburgh, Scotland).

[48] Kondrup J, Johansen N, Plum LM, Bak L, Larsen IH, Martinsen A (2002). Incidence of nutritional risk and causes of inadequate nutritional care in hospitals. Clin Nutr (Edinburgh, Scotland).

[49] Dupertuis YM, Kossovsky MP, Kyle UG, Raguso CA, Genton L, Pichard C (2003). Food intake in 1707 hospitalised patients: a prospective comprehensive hospital survey. Clin Nutr (Edinburgh, Scotland).

[50] Wischmeyer PE (2017). Tailoring nutrition therapy to illness and recovery. Crit Care (London, England).

[51] Rice TW, Wheeler AP, Thompson BT, Steingrub J, Hite RD, Moss M (2012). Initial trophic vs full enteral feeding in patients with acute lung injury: the EDEN randomized trial. JAMA.

[52] Braunschweig CA, Sheean PM, Peterson SJ, Gomez Perez S, Freels S, Lateef O (2015). Intensive nutrition in acute lung injury: a clinical trial (INTACT). JPEN J Parenter Enter Nutr.

[53] Arabi YM, Aldawood AS, Haddad SH, Al-Dorzi HM, Tamim HM, Jones G (2015). Permissive underfeeding or standard enteral feeding in critically Ill adults. N Engl J Med.

[54] Alberda C, Gramlich L, Jones N, Jeejeebhoy K, Day AG, Dhaliwal R (2009). The relationship between nutritional intake and clinical outcomes in critically ill patients: results of an international multicenter observational study. Intensiv Care Med.

[55] Wischmeyer PE, Hasselmann M, Kummerlen C, Kozar R, Kutsogiannis DJ, Karvellas CJ (2017). A randomized trial of supplemental parenteral nutrition in underweight and overweight critically ill patients: the TOP-UP pilot trial. Crit Care (London, England).

[56] Fuentes Padilla P, Martínez G, Vernooij RW, Urrútia G, Roqué I Figuls M, Bonfill Cosp X (2019). Early enteral nutrition (within 48 hours) versus delayed enteral nutrition (after 48 hours) with or without supplemental parenteral nutrition in critically ill adults. Cochrane Database Syst Rev..

[57] Yeh DD, Johnson E, Harrison T, Kaafarani HMA, Lee J, Fagenholz P (2018). Serum levels of albumin and prealbumin do not correlate with nutrient delivery in surgical intensive care unit patients. Nutr Clin Pract.

[58] Davis CJ, Sowa D, Keim KS, Kinnare K, Peterson S (2012). The use of prealbumin and C-reactive protein for monitoring nutrition support in adult patients receiving enteral nutrition in an urban medical center. JPEN J Parenter Enter Nutr.

[59] Bear DE, Griffith D, Puthucheary ZA (2018). Emerging outcome measures for nutrition trials in the critically ill. Curr Opin Clin Nutr Metab Care.

