# Supplementary material for: Malnutrition and its effects in severely injured trauma patients
Source: Eur J Trauma Emerg Surg. 2020 Jan 23;46(5):993–1004. doi: 10.1007/s00068-020-01304-5 (PMC7593306; doi:10.1007/s00068-020-01304-5)
Supplement: Supplementary file 1 — Supplementary file1 (DOCX 16 kb) [file 68_2020_1304_MOESM1_ESM.docx]

**Malnutrition and its effects in severely injured trauma patients**

*Suzan Dijkink*^1^ MD, Karien Meier*^1^ MSc*, Pieta Krijnen^1^ PhD, D. Dante Yeh^2^ MD, George C. Velmahos^3^ MD PhD,
Inger B. Schipper^1^ MD PhD

*^*S. Dijkink and K. Meier equally contributed to this manuscript.^*

^1 Department of Surgery, Leiden University Medical Center, Leiden, The Netherlands
2 Ryder Trauma Center, DeWitt Daughtry Family Department of Surgery, University of Miami Miller School of Medicine, Miami, Florida, USA.
3 Division of Trauma, Emergency Surgery, and Surgical Critical Care, Department of Surgery, Massachusetts General Hospital, Boston, Massachusetts, USA^

**Corresponding author**

Suzan Dijkink MD

Department of Trauma Surgery

Leiden University Medical Center, K6-R
P.O. Box 9600
2300 RC Leiden

Fax number: +3171 526 6750

Phone number: +3171 5261367

[S.Dijkink@lumc.nl](mailto:S.Dijkink@lumc.nl)

**Appendix I.** Search strategy.

| **Database** | **Search strategy** |
| --- | --- |
| **PubMed** | ("Nutritional Status"[Mesh] OR "Nutritional Status"[tw] OR "Nutrition Status"[tw] OR "nutrition state"[tw] OR "nutritional state"[tw] OR "Malnutrition"[Mesh] OR "Malnutrition"[tw] OR "Nutritional Deficiency"[tw] OR "Nutritional Deficiencies"[tw] OR "Undernutrition"[tw] OR "Malnourished"[tw] OR "Malnourishment"[tw] OR "Malnourishments"[tw] OR "fed state"[tw]) AND ("Multiple Trauma"[Mesh] OR "Multiple Trauma"[tw] OR "Multiple Traumas"[tw] OR "polytrauma"[tw] OR "polytraumatized"[tw] OR "polytraumatised"[tw] OR "multitrauma"[tw] OR "poly-trauma"[tw] OR "multi-trauma"[tw] OR "polytraumatic"[tw] OR "multitraumatic"[tw] OR "poly-traumatic"[tw] OR "multi-traumatic"[tw] OR "Multiple Injury"[tw] OR "Multiple Injuries"[tw] OR "Wounds and Injuries"[Mesh:NoExp] OR "trauma"[tw] OR "traumas"[tw] OR "traumatic"[tw] OR "Critical Illness"[Mesh] OR "Critical Illness"[tw] OR "critically ill"[tw] OR "intensive care"[tw] OR "Critical Care"[Mesh] OR "Critical Care"[tw]) NOT ((child NOT child[au]) OR child*[tw] OR children*[tw] OR schoolchild*[tw] OR infan*[tw] OR pediatri*[tw] OR paediatr*[tw] OR neonat*[tw] OR boy[tw] OR boys[tw] OR boyhood[tw] OR girl[tw] OR girls[tw] OR girlhood[tw] OR youth[tw] OR youths[tw] OR baby[tw] OR babies[tw] OR toddler*[tw] OR teen[tw] OR teens[tw] OR teenager*[tw] OR newborn*[tw] OR postneonat*[tw] OR postnat*[tw] OR puberty[tw] OR preschool*[tw] OR suckling*[tw] OR picu[tw] OR nicu[tw] OR juvenile[tw]) |
| **Embase** | (exp *nutritional status/ OR "Nutritional Status".ti. OR "Nutrition Status".ti.  OR "nutrition state".ti. OR "nutritional state".ti. OR exp *Malnutrition/ OR "Malnutrition".ti. OR "Nutritional Deficiency".ti. OR "Nutritional Deficiencies".ti. OR "Undernutrition".ti. OR "Malnourished".ti. OR "Malnourishment".ti. OR "Malnourishments".ti. OR "fed state".ti.) AND (exp *"multiple trauma"/ OR "Multiple Trauma".ti. OR "Multiple Traumas".ti. OR "polytrauma".ti. OR "polytraumatized".ti. OR  "polytraumatised".ti.  OR "multitrauma".ti. OR "poly-trauma".mp. OR "multi-trauma".mp. OR "polytraumatic".mp. OR "multitraumatic".mp. OR "poly-traumatic".mp. OR "multi-traumatic".mp.OR "Multiple Injury".ti. OR "Multiple Injuries".ti. OR "trauma".ti. OR "traumas".ti. OR "traumatic".ti. OR exp *"Critical Illness"/ OR "Critical Illness".ti. OR "critically ill".ti. OR "intensive care".ti.OR *intensive care/ OR "Critical Care".ti.) |
